# Nutritional management of pediatric nephrotic syndrome regarding oxidative stress and antioxidant balance

**DOI:** 10.3389/fimmu.2025.1542735

**Published:** 2025-05-01

**Authors:** Elena Jechel, Alin Horatiu Nedelcu, Felicia Dragan, Vasile Valeriu Lupu, Iuliana Magdalena Starcea, Adriana Mocanu, Solange Tamara Rosu, Violeta Streanga, Ruxandra Russu, Ginel Baciu, Ciprian Danielescu, Delia Lidia Salaru, Ionela Daniela Morariu, Olga Cirstea, Emil Anton, Ancuta Lupu

**Affiliations:** ^1^ Pediatrics, Faculty of Medicine, “Grigore T. Popa” University of Medicine and Pharmacy, Iasi, Romania; ^2^ Faculty of Medicine, “Grigore T. Popa” University of Medicine and Pharmacy, Iasi, Romania; ^3^ Faculty of Medicine and Pharmacy, University of Oradea, Oradea, Romania; ^4^ Pediatrics, Faculty of Medicine and Pharmacy, “Dunarea de Jos” University of Galati, Galati, Romania; ^5^ Faculty of Pharmacy, “Grigore T. Popa” University of Medicine and Pharmacy, Iasi, Romania; ^6^ Pediatrics, “Nicolae Testemitanu” State University of Medicine and Pharmacy, Chisinau, Moldova

**Keywords:** oxidative stress, child, kidney damage, systemic progression, nutritional management

## Abstract

Pediatric nephrotic syndrome remains a complex clinical entity, with incompletely elucidated pathogenetic mechanisms, in which oxidative stress appears to have a substantial etiopathogenic role. Recent evidence supports the involvement of redox imbalance in podocyte damage, impaired glomerular function, and systemic decline. All this suggests that antioxidant interventions can favorably modulate the course of the disease. This narrative review aims to synthesize the most relevant data from the current literature on the interaction between oxidative stress and nephrotic syndrome in children, with a focus on the therapeutic potential of antioxidants. The analysis focuses on the molecular mechanisms by which oxidative stress contributes to the progression of renal dysfunction, the role of oxidative biomarkers in disease monitoring, and the ability of antioxidants to reduce the need for immunosuppressants and corticosteroids, thus contributing to the decrease in associated morbidity. The translational perspectives of antioxidant therapy are also discussed, in the context of the urgent need for effective adjuvant strategies with a safety profile superior to conventional therapies. By integrating these data, the paper supports the valorization of antioxidant interventions as an emerging direction in the management of pediatric nephrotic syndrome and substantiates the need for controlled clinical trials, with rigorous design, in this field.

## 1 Introduction

Nephrotic syndrome (NS) is a complex condition characterized by a chronic undulating evolution and is associated with increased morbidity and mortality. Particularly concerning pediatric patients, its individualized, multidisciplinary management is vital to mitigate systemic decline and improve both morbidity and psycho-social well-being (1, 2). Clinically, NS presents with proteinuria (urinary protein/creatinine ratio over 24 hours ≥ 200 mg/mmol or 3+ on urine strips), hypoalbuminemia (serum albumin < 3 g/dl), and edema. Additionally, patients with NS can also exhibit hyperlipidemia and an increased risk of thrombosis (3, 4). The incidence and prevalence of pediatric NS are approximately 3 per 100,000 and 16 per 100,000 children respectively, with a peak occurrence between ages 2 and 7. Genetic predisposition and geographic factors contribute significantly to disease distribution. Multiple genetic variations have been identified in intrafamilial aggregates and NS appears to be more common among young boys (5–7).

NS can be divided into primary and secondary forms. Secondary NS arises as a consequence of other systemic pathologies (e.g., infections, neoplasms, systemic lupus erythematosus, diabetes) or the improper use of nephrotoxic substances (e.g., medications) (8, 9). A rarer form, congenital NS is defined as the appearance of symptoms in newborns or infants younger than 3 months and is primarily linked to genetic variations, perinatal infections (e.g. syphilis, toxoplasmosis), or alloimmune diseases (10, 11). Among these, primary NS is the most prevalent in pediatrics. Depending on the histopathological characteristics of kidney damage, NS most commonly presents with minimal lesions or as focal segmental glomerulosclerosis (FSGS) (8). In addition to the microscopic aspect, the minimal lesions appear earlier (median age of 3 years versus 6 years in FSGS) and respond more favorably to corticosteroid treatment. In contrast, current data suggests that FSGS responds poorly to steroids and is therefore an important cause of progression to chronic kidney disease in children (6, 8, 12).

If a patient is unresponsive to steroid treatment, the current guidelines recommend escalating therapy by combining immunosuppressive drugs such as calcineurin inhibitors (Cyclosporin, Tacrolimus), mycophenolate mofetil, anti-CD20 monoclonal antibodies (Rituximab, Ofatumumab), and CTLA- 4 (Abatacept, Belatacept). The preference for a certain type of medication remains dictated by the particularities of the patient (12–15). To prevent and counteract the main comorbidities specific to NS, supportive therapy must include lifestyle modifications (e.g., diet) or the administration of pharmacological substances such as diuretics, lipid-lowering drugs, antihypertensives (renin-angiotensin axis inhibitors), and anticoagulants (8, 12, 16). Current global research highlights the decisive role of intestinal microbiota modulation through the intestinal axes - target organ system. Studies note that microbiome dynamics can negatively impact the prevalence and clinical course of various pathologies, including inflammatory, autoimmune, and atopic diseases. Among the physiological mechanisms affected in this process, we note the induction of chronic low-grade systemic inflammation and an imbalance in oxidative status, marked by increased oxidative stress (OS) and reduced total antioxidant capacity (17–20). The interrelationship between the two (increased OS, together with decreased antioxidant capacity) induces a systemic deficiency in endogenous antioxidant (EA) defenses (e.g., glutathione, superoxide dismutase, catalase) which aggravates cellular damage, promotes podocyte damage, and increases proteinuria. This sustains the loss of urinary albumin, a protein with antioxidant properties, while maintaining the vicious cycle of inflammation, glomerular injury, and endothelial dysfunction. The effect of proteinuria on urinary excretion of microelements in NS has been intensively studied, revealing significantly higher urinary losses in children experiencing relapse. This loss is accompanied by a blood deficiency of these microelements compared to children in remission and healthy controls. Over time, these factors contribute to the disruption of systemic and renal homeostasis, leading to NS-specific complications, progression to chronic kidney disease, and a diminished capacity for optimal treatment response. Additionally, the literature highlights a bidirectional correlation between oxidative status and therapeutic response/efficacy. Zinc has been identified as a promising adjuvant in the treatment of pediatric NS, with supplementation helping to reduce relapses and infection-related episodes associated with relapse, thereby prolonging the disease-free interval. A decrease in pro-inflammatory status parallels the development of remission, while oxidative and nitrative stress influence the response to steroid therapy. This relationship is reinforced by the strong link between selenium deficiency – resulting from urinary albumin-induced losses – and steroid resistance. The latter may occur possibly through the oxidation and inactivation of glucocorticoid receptors, coupled with reduced receptor expression at the cellular level. Selenium also intervenes in the dynamics of the malondialdehyde-glutathione balance and total antioxidant capacity. Consequently, we are confident that reducing OS markers in children with NS will enhance therapeutic response (21–30).

Based on these findings and current trends, this work intends to showcase the current understanding of how exogenous (ExA) and EAs influence the progression of pediatric NS. We will look at data on the absorption and secretion of antioxidants and antioxidant-related components, physiological metabolism, and the pathological pathways that arise when internal homeostasis is disrupted. To meet our goal, we performed a comprehensive literature review without setting strict inclusion criteria. We sourced the most recent available data from PubMed, Google Scholar, Web of Science (WoS), Scopus, and Embase, including subject encyclopedias. Search criteria were, therefore, defined by using terms and phrases like: “*Nephrotic Syndrome*”, “*Renal Damage*”, “*Chronic Renal Progression*”, combined with “*Child*”, “*Oxidative Stress*”, “*Endogenous Antioxidants*”, “*Exogenous Antioxidants*”, “*Prognostic Biomarkers*”, “*alternative therapies*”, and “*Individualized management*”. The study population included patients younger than 18 years at the time of reporting. To address gaps in the pediatric literature, we supplemented our analysis with relevant findings from adult studies and murine models where necessary. We minimized the risk of bias by not restricting our search based on language. Through this research, we aim to expand and integrate knowledge about oxidative balance in NS dynamics, considering major pathophysiological mechanisms and their implications. Ultimately, we hope our findings will contribute to advancing the individualized diagnosis and management of pediatric NS.

## 2 Main pathophysiological mechanisms involved in NS

At the level of the renal interstitium, the boundary between the glomerular capillaries and the urinary space is formed by the filtration membrane, which serves as a dual structural and electrochemical barrier. This membrane is made up of the capillary endothelium, the glomerular basement membrane, and podocyte extensions. Its main role is to stop the passage of substances with high molecular weight (e.g., proteins) into the urine. In NS, the glomerular basement membrane undergoes structural changes that alter its mechanical function and electrostatic balance. The main changes are reflected in the podocyte structures, the essential element in the glomerular filtration barrier. Notably, the disappearance of the slit diaphragm and the erasure of podocytic processes were observed (31–33). Following these changes, the injured glomerular filtration membrane allows the passage of albumin molecules (selective proteinuria) or high molecular weight plasma proteins (non-selective proteinuria) into the urine. Among the proteins with a functional role lost in the case of non-selective proteinuria, we note transferrin, ceruloplasmin, immunoglobulins, and fractions of the complement system, factors involved in the dynamics of coagulation, but also other enzymes and vitamins with a role in various metabolic processes (31, 34–36).

Persistent proteinuria contributes to progressive renal impairment, ultimately leading to the destruction of physiological architecture, tubular atrophy, and interstitial fibrosis (37, 38). Among the clinical-biological consequences of the pathological glomerular disturbances encountered in NS, we note malnutrition, anemia, nutritional deficiencies, procoagulant status, hypoalbuminemia, dyslipidemias, endocrinopathies, and increased susceptibility to infections due to immune deficiency. These, together with other such examples, are presented in a centralized manner in **Table 1**. The entire hemodynamic balance is deregulated by the activation of the renin-angiotensin system, the occurrence of hydro-electrolytic and acid-base imbalances, and peripheral edema, localized or generalized, which affect vital organs such as the nervous system, osteoarticular apparatus, liver, lung parenchyma, and cardiovascular system (38–42). This process perpetuates a vicious cycle of renal deterioration, characterized by hyperfiltration, reduced arteriolar resistance, compensatory glomerular hypertrophy, and progressive systemic decline (35, 36).

**Table 1:** Main complications in the evolution of NS (40, 41).

Type of complication	Complication	Mechanism	Management
Caused by disease	Infections	Deficiency of immunoglobulin G, factor B and I of the alternative pathway, transferrin. T cell dysfunction. Decreased humoral defense capacity following edema.	Vaccination Antiviral therapy Administration of Ig in case of exposure to varicella zoster virus.
	Thromboembolism	Platelet abnormalities Pro-coagulant status through increased synthesis of factors V, VII, VIII, X, von Willebrand factor, fibrinogen and α2-macroglobulin accumulation Decreased activity of endogenous anticoagulants: antithrombin III, protein C, protein S and tissue factor pathway inhibitor Decreased activity of the fibrinolytic system Changes in the glomerular hemostatic system Intravascular volume depletion Exposure to corticosteroids and diuretics	Avoiding arterial puncture Evaluation by Doppler ultrasound/magnetic resonance angiography in the case of macroscopic hematuria with/without renal failure Ventilation-perfusion lung scan/pulmonary angiography in case of tachypnea and dyspnea.
	Hypovolemia	Hypoalbuminemia Vomiting High doses of diuretics	Intravenous administration of normal saline (20 mL/kg over 1 to 2 hours) or albumin at a maximum dose of 1 g/kg over 3 to 5 hours with blood pressure monitoring
	Cardiac damage	Endothelial dysfunction Hyperlipidaemia Pro-thrombotic status	Administration of lipid-lowering drugs, hydroxy-methyl-glutaryl coenzyme A reductase inhibitors - controversial in pediatrics
	Anemia	Iron deficiency Urinary losses of transferrin	Correction of proteinuria Administration of iron preparations Administration of recombinant erythropoietin
	Acute renal failure	Massive proteinuria, hypoalbuminemia and reduced plasma volume induce circulatory collapse or prerenal uremia. Other causes such as septicemia, contrast media, post-antibiotic acute tubular necrosis, and nonsteroidal anti-inflammatory agents may precipitate renal injury.	Fluid loss replacement Diuretic therapy In case of unresponsiveness - dialysis.
	Edema	Increase in glomerular permeability and hypoalbuminemia, causes a decrease in oncotic pressure, functional hypovolemia and stimulates secondary sodium retention by the kidneys.	Sodium restriction Diuretics Albumin
	Hormonal dysfunctions	Thyroid involvement: urinary loss of hormone-binding proteins. Hypocalcemia: decreased albumin level, increased renal tubular reabsorption, abnormalities in vitamin D metabolism.	Thyroid screening and subclinical mineral bone diseases Early substitution if necessary
Caused by drugs	Obesity, growth retardation, hypertension, osteopenia, avascular necrosis of the femoral head, cataract, glaucoma, emotional disorders.	Corticosteroids Additional: loss of insulin-like growth factors and/or IGF-binding proteins.	Adrenal suppression: alternate-day steroid therapy. Impairment of stature: steroids sparing agents and growth hormone therapy. Osteoporosis: Calcium supplementation, vitamin D, and use of steroid-sparing protocols. Peptic ulceration: H2 blockers. Hypertension: antihypertensive agents. Cataract: low dose and short duration of steroids treatment, regular examination by ophthalmologists. Increased intracranial pressure: investigate papilledema. Behavioral changes: reduce or withdraw steroids.
	Bone marrow suppression, alopecia, nausea, vomiting, hemorrhagic cystitis, infections, infertility, secondary malignancy.	Alkylating agents (e.g., Cyclophosphamide) Supplement: can produce gonadal toxicity. Not to be used for more than 12 weeks (2 mg/kg, single oral dose) Stop if the white blood cell count is less than 5,000/mm3	Hemorrhagic cystitis: high water intake.
	Nephrotoxicity, neurotoxicity, gingival hyperplasia, hirsutism, hypertension.	Cyclosporin A Risk factors for complications: long duration of treatment, high dose, young age at the start of treatment.	It is recommended to use the lowest effective dose for maintenance treatment, tapering over a year to 1–3 mg/kg/day.
	Nausea, vomiting, bone marrow suppression.	Mycophenolate mofetil	Dose reduction or discontinuation in severe cases.
	Diabetes, hypertension, nephrotoxicity, tremor, headache, hyperkalemia, hypophosphatemia, leukopenia.	Tacrolimus	–
	Bronchospasm, myocardial infarction, progressive multifocal leukoencephalopathy, reactivation of viruses (e.g., cytomegalovirus, hepatitis B virus).	Rituximab	–

**Figure 1:**
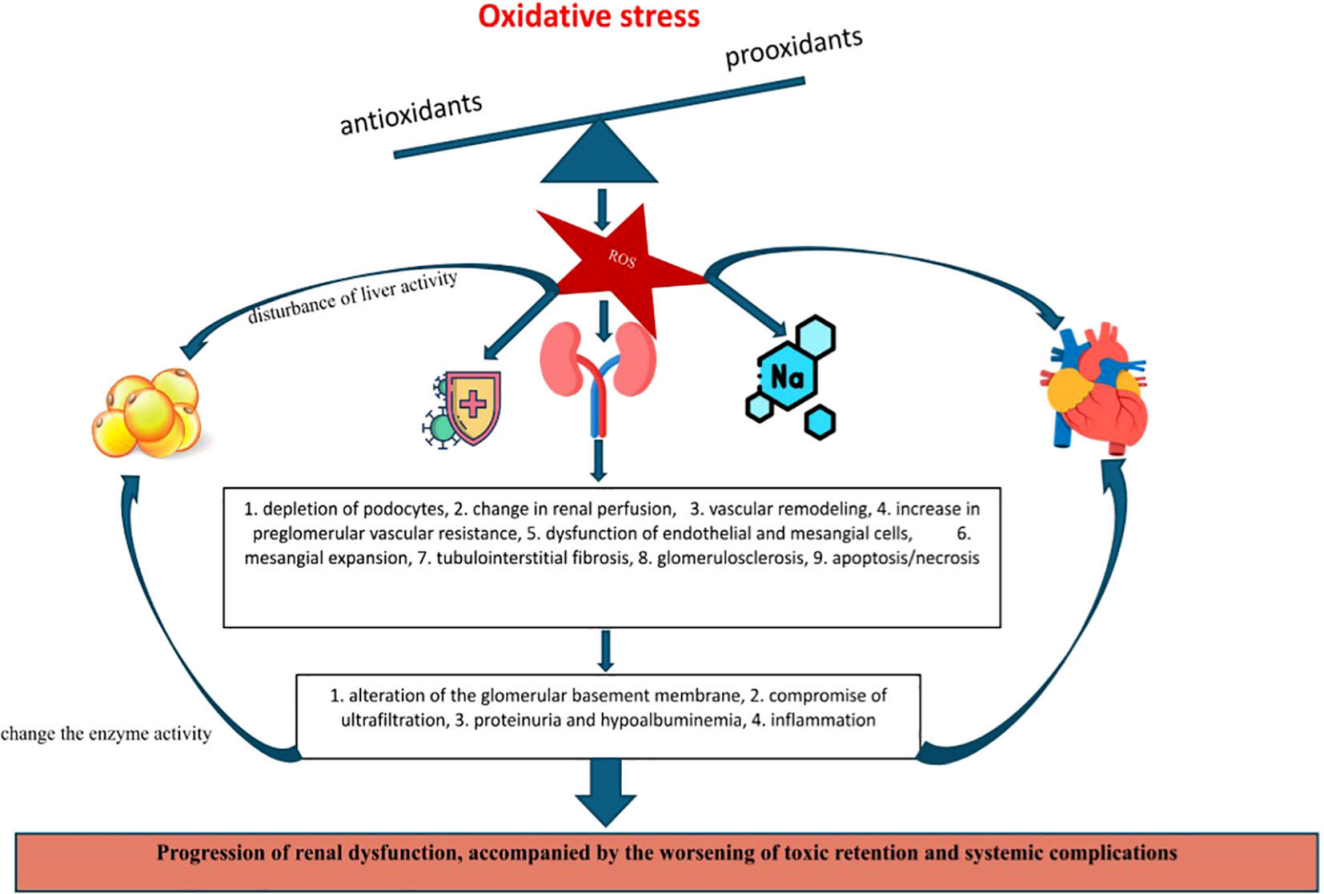
OS implications in the promotion of renal dysfunction and specific complications of pediatric NS.

In the extensive process described previously, the systemic pro-oxidative status appears to be the central driver of the vicious cycle. The increase in free radicals together with the decrease in antioxidant capacity both play a pivotal role in potentiating renal damage by causing damage to the glomerular filtration barrier, cell damage, and endothelial dysfunction. Aside from this, they also promote inflammation, disrupt lipid metabolism, alter the optimal therapeutic response, or favor the development of comorbidities - aspects observed in the pediatric population, but also in adults. The pro-oxidant – antioxidant balance has also been shown to correlate with the evolutionary stages of NS (43–45). Over time, dyslipidemia and OS become critical factors in atherosclerosis development and the progression to cardiovascular disease and chronic renal failure. In support of this, Jabarpour M. et al. emphasize the importance of implementing therapies to mitigate these two variables (46). Furthermore, Bufna A. et al. report that corticosteroid induction therapy appeared to improve total antioxidant capacity (47)., However, caution is warranted, as other studies report that markers of OS remain elevated even after steroid therapy, highlighting its persistent role in NS pathophysiology (45). Consequently, its implications in NS dynamics remain indisputable, being detailed in what follows. Finally, **Figure 1** schematically illustrates the pathophysiological cascade specific to the onset, progression, and complication of NS in pediatrics with respect to OS.

## 3 Oxidative stress

OS is defined as a disturbance of the systemic pro-oxidative - anti-oxidative balance. The consequences of this imbalance are the excessive production of reactive oxygen species (ROS), reactive nitrogen species, and other species of free radicals. At the same time, there is a reduced antioxidant capacity to neutralize reactive species and counteract damage. Physiologically, OS has a role in cell signaling and immune defense. But, when its level exceeds the capacity of antioxidant defense, reactive species can induce the oxidation of lipids, proteins, and nucleic acids. These seem to be the mechanisms by which ROS participate in the induction of low-grade chronic inflammation, cellular dysfunction, and implicitly in the promotion and aggravation of chronic conditions (e.g., cardiovascular, pulmonary, renal, metabolic, neurological, malignancies) (48, 49). Following on from what has been discussed, Bissinger R. et al. note that OS acts as a bridge between chronic conditions and various complications such as anemia (50).

Regarding kidney damage, OS is correlated in the literature with both antioxidant depletion and excessive ROS production, serving as both a cause and an effect of renal degradation. The kidney’s vulnerability to OS appears to stem from its intense metabolic activity at the mitochondrial level. In relation to this, Daenen K. et al. reassert the link between OS and the progression of renal damage, further complicated by hypertension, atherosclerosis, inflammation, and anemia (51). In particular, cardiovascular damage is greatly accentuated in patients undergoing replacement therapy, such as hemodialysis, peritoneal dialysis, or renal transplantation, where persistent exposure to a pro-oxidative state intensifies systemic deterioration (52).

Given the complexity of this systemic condition, **Table 2** summarizes the main exogenous and endogenous factors that interfere with the oxidative balance in kidney damage. To counteract their effects and restore homeostasis, the presence of enzymatic and non-enzymatic antioxidants in circulation is necessary. These antioxidants are currently recognized as important both in expanding diagnostic capabilities but also for their therapeutic role in mitigating systemic damage (53, 54).

**Table 2:** Main sources of oxidative stress identified in kidney damage (55–64).

Source	Substrate	Mechanism	Implications
Endogenous	Mitochondria	Oxidative phosphorylation represents a potent source of electrons which, in combination with molecular oxygen, forms the superoxide anion - substrate for the formation of hydrogen peroxide and the hydroxyl radical.	Directly: the apoptosis of renal cells caused by the oxidation of lipids, proteins and nucleic acids leads to the deterioration of nephron function with the subsequent progression of NS. Indirectly: chronic inflammation maintains glomerular fibrosis and sclerosis.
	NADPH oxidase	Pro-inflammatory stimuli and hyperglycemia intensify the activity of NADPH oxidase, the enzyme involved in the formation of superoxide anion.	It intervenes in the damage of podocytes and the glomerular basement membrane, accentuating proteinuria. It affects the normal structure of the kidney by activating myofibroblasts, depositing the extracellular matrix and stimulating fibrosis.
	Xantin oxidase	Enzyme expressed at the level of the vascular endothelium and in the renal epithelial cells which, under conditions of hypoxia due to ischemia or reperfusion, intensifies the generation of superoxide anion and hydrogen peroxide.	It maintains ischemia by oxidizing lipids and damaging nucleic acids, promoting acute tubular necrosis. It promotes inflammation and apoptosis, contributing to the progression of the disease.
	Cyclooxygenase (COX)	Overexpression of COX-2 due to renal inflammation induces an increase in the generation of secondary products of enzymatic activity such as ROS.	It exacerbates proteinuria by damaging the glomerular filtration barrier and increasing vascular permeability. It favors endothelial dysfunction and vasoconstriction by reducing the bioavailability of nitric oxide.
	Lipid peroxidation	It intervenes in the destruction of cell and mitochondrial membranes.	Amplifies cell damage and apoptosis.
	Cytochrome P450	ROS are generated secondary to the metabolism of toxic substances or drugs.	Potentiates medicinal nephrotoxicity. It induces exaggerated inflammatory responses, collagen deposition and accentuates renal fibrosis.
	Neutrophils	Chronic inflammation is accompanied by the continuous recruitment of neutrophils that generate ROS following the inflammatory response directed towards the destruction of pathogens.	It exacerbates the loss of kidney function by damaging the normal architecture. It maintains the inflammatory process and renal damage by recruiting and activating other immune cells.
Exogenous	Environmental pollutants	Direct or indirect generation of ROS, either through interaction with the molecular substrate of cells, or through the activation of specific metabolic pathways. Inhibiting the activity of endogenous antioxidant enzymes (e.g. superoxide dismutase, catalase, glutathione peroxidase. Potentiation of chronic inflammation, ROS production and tissue damage.	They maintain glomerular and tubular damage, fibrosis and alteration of normal renal architecture. Exacerbates proteinuria. They induce endothelial dysfunction, vasoconstriction and hypertension. They can negatively interfere with the effectiveness of the treatment.
	Active or passive smoking		
	Diet		
	Medicines and chemicals		
	Ultraviolet and ionizing radiation		

## 4 Consequences of oxidative stress in renal and systemic decline

Summarizing the above, we note that OS (whether exogenous or endogenous) serves an important role in promoting and sustaining of systemic damage. This is mainly achieved by maintaining a chronic inflammatory status that disrupts physiological molecular processes. In the renal system, OS has been implicated both in renal injury and in increasing the risk of complications, including impaired therapeutic responses (e.g., corticosteroid resistance). The following sections provide a detailed exploration of these aspects and the pathophysiological mechanisms that amplify their impact.

### 4.1 Renal and glomerular injuries

Optimal renal functions – such as waste filtration, regulation of electrolyte balance, and bone integrity maintenance – are closely linked to adequate circulatory flow. It is estimated that the kidneys receive approximately one-fifth of cardiac output at rest, yet the oxygen pressure in the renal interstitium remains relatively low due to the arterio-venous mechanism. Notably, the partial pressure of O2 is higher in the renal vein than in the efferent arteriole or cortex. Renal blood flow is regulated by both intrinsic factors (e.g., myogenic response, tubulo-glomerular feedback) and extrinsic factors (e.g., neuro-hormonal signals). These self-regulatory changes in the renal plasma flow are intended to minimize the variations in the ultrafiltered volumes (65–68). Under conditions of oxidative stress, reactive species can alter renal perfusion, leading to vascular remodeling and increased preglomerular resistance – key factors in the development of hypertension as well as acute and chronic renal lesions. The primary target of this process is the tone of the renal afferent arteriole, followed by alterations in the myogenic response and tubulo-glomerular feedback (68, 69).

At the cellular level, OS represents a significant cause of endothelial and mesangial cell dysfunction, which in turn compromises vascular and glomerular integrity. Under normal conditions, endothelial cells promote vasodilation by facilitating the release of vasoactive substances. However, excess ROS interact with nitric oxide to form peroxynitrite, reducing nitric oxide bioavailability. Given nitric oxide’s critical role in vasodilation, anti-aggregation, and leukocyte adhesion prevention, its depletion leads to endothelial dysfunction. Subsequently, the activated endothelial cells produce vasoconstrictor agents, amplifying the inflammatory response. The resulting upregulation of adhesion molecules and secretion of chemokines establishes a vicious cycle that sustains inflammation (70).

The reference studies attest that endothelial dysfunction appears from the first stages of renal damage and correlates with disease activity, particularly the degree of proteinuria and serum albumin levels. Paraclinically, markers such as soluble thrombomodulin, tissue plasminogen activator, plasminogen activator inhibitor-1, and von-Willebrand factor help to assess this dysfunction. Endothelial damage not only contributes to systemic atherosclerosis but also accelerates kidney disease progression by increasing capillary permeability and aggravating proteinuria. However, evidence suggests that this process may be reversible, making it a potential therapeutic target for mitigating renal injury (71–73).

Further studies on chronic kidney damage models, such as diabetic kidney disease, reveal that podocyte depletion occurs due to the exposure of glomeruli to harmful stimuli, OS inducers such as hyperglycemia, hyperuremia, increased levels of fatty acids, growth factors, cytokines, and hormones (74–76). OS-induced podocyte damage manifests in multiple ways, primarily through the disruption of structural and functional integrity. This includes membrane dysfunction, cytoskeletal damage, mitochondrial impairments, inefficient energy production, as well as the activation of pro-apoptotic and pro-necrotic signaling pathways (e.g., caspases, mitogen-activated protein kinase). Other mechanisms involved in OS-induced podocyte damage include autophagy, pyroptosis, ferroptosis, and mitotic catastrophe.

In this sense, it is noted that targeting cellular pathways involved in OS dynamics (e.g., modulation of the TSP-1/TGF-β1/Smad pathway by overexpression of Sestrin2) protects podocytes from apoptosis. Conversely, exposure to harmful stimuli (e.g., hyperglycemia) has been associated with decreased protective autophagy. Thus, current efforts are focused on preserving basal autophagy levels in podocytes, a goal achievable through the administration of antioxidant compounds such as berberine, mangiferin, notoginsenoside R1, and geniposide (75, 77, 78). From a pathophysiological perspective, OS-induced podocyte apoptosis and necrosis compromise the glomerular filtration barrier, impairing ultrafiltration. This disruption allows plasma proteins to pass through the glomerular barrier, depending on their size and extent of the damage. The subsequent proteinuria perpetuates a vicious cycle by triggering inflammatory responses which further exacerbate OS, worsening podocyte damage and sustaining NS-specific symptomatology – possibly through the activation of the Wnt/β-catenin pathway (77–81).

Excessive OS exerts a dual impact on the integrity of the glomerular filtration barrier, both inducing dysfunction and exacerbating proteinuria and renal decline. Research highlights a bidirectional relationship between glomerular endothelial dysfunction following OS and podocyte damage, reflected in the dynamics of vascular endothelial growth factor A, angiopoietins, CXCL12/CXCR4/CXCR7, endothelin-1, interleukin (IL)-6, and extracellular vesicles at the paraclinical level. In practical terms, endothelial dysfunction disrupts glomerular pressure and blood flow, exacerbating local ischemia and the pro-oxidative status, accelerating podocyte damage. In turn, damaged podocytes lose their ability to maintain glomerular structure and trigger a local inflammatory reaction. It is also noted that counteracting mitochondrial OS by means of ExAs has led to the prevention of podocyte loss, albuminuria, and glomerulosclerosis, improving renal function. Findings from murine models suggest that early renal damage is characterized by endothelial cell injury, which precedes podocyte process deletion (74, 75, 77, 80, 82).

### 4.2 Inflammation and fibrosis

Following renal parenchymal damage due to OS, repair and regeneration processes are initiated. Physiologically, the repair of damaged tissue is characterized in the first phase by the appearance of myofibroblasts which contribute to the production of collagen and other components of the extracellular matrix. The success of parenchymal regeneration depends on the integrity of this process. Pathological variations occur when inflammatory and fibrogenic signaling is either incomplete or persistently activated. In the former case, repair remains latent and seems to re-initiate after an acute insult. In the latter, excessive extracellular matrix deposition leads to structural and functional impairment, culminating glomerular sclerosis (83).

Nuclear factor kappa-light-chain-enhancer of activated B cells (NF-κB) plays an important regulatory role in the expression of genes involved in inflammation, immune response, and cell survival. Under physiological conditions, NF-κB is located in the cytosol in an inactive state, bound to inhibitory proteins. Upon stimulation by viral or bacterial antigens, cytokines, or growth factors, NF-κB activation is transient, lasting approximately 30–60 minutes. It is noted that, under the conditions of a prolonged pro-inflammatory status, ROS can activate kinases such as the IKK complex (composed of two kinases, IKKα and IKKβ, and the regulatory subunit NEMO/IKKγ). In response to this interaction, IKK phosphorylates inhibitory κB proteins at the level of two critical serine residues. They are subsequently degraded in the proteasome and translocated into the nucleus where they bind to specific DNA sequences and initiate the transcription of target genes involved in the immune response, inflammation (e.g., tumor necrosis factor (TNF)-α, ILs 1 and 6, prostaglandins), chemotaxis (e.g., IL-8), cell adhesion (intercellular adhesion molecule-1, vascular adhesion molecule-1), growth regulation, and apoptosis resistance. In turn, cytokines and chemokines attract and activate inflammatory cells (neutrophils, macrophages, T lymphocytes) within the renal tissue, amplifying the chronic inflammatory response and perpetuating NF-κB activation (84–87). Thus, NF-κB inhibition through antioxidants has been proposed as a therapeutic target in chronic inflammatory conditions, including atherosclerosis and OS-induced nephrotoxicity (87, 88). In addition to stopping the chronic inflammatory feedback loop, Udwan K. notes that targeting Nf-κB appears to be involved in preventing changes in water permeability and protein reflection coefficient, thereby reducing ascites (89).

Furthermore, chronic inflammation leads to the progressive deterioration of renal function by promoting and maintaining fibrosis. This interplay is mediated primarily by transforming growth factor β1 (TGF-β1). By stimulating the TGF-β1/Smad pathway and interacting with surface receptors, numerous signaling pathways are activated that ultimately result in the activation of myofibroblasts, either directly from interstitial fibroblasts or through the trans-differentiation of endothelial cells or mesangial epithelial cells. Connective tissue growth factor enhances this process by improving the affinity of TGF-β for its receptors, thereby sustaining fibrogenic activity. Subsequently, the myofibroblasts excessively stimulate the production of collagen and components of the extracellular matrix, leading to tubulointerstitial fibrosis, mesangial expansion, and glomerulosclerosis. This pathological cascade has emerged as a therapeutic target for antioxidant-based interventions, particularly those based on activators of nuclear factor erythroid 2-related factor 2, which aim to reduce fibrosis, senescence, and apoptosis while enhancing regeneration capacity (90–93). Additional pathways implicated in tubulointerstitial injury and renal fibrosis include TNF-α and tissue inhibitor of metalloproteinase mRNA-1, which facilitate fibrosis by preventing the breakdown of collagen (93). Finally, the Jun N-terminal kinase signaling pathway interacts with the TGF-β1/Smad pathway, establishing its role in fibrotic progression (94). Additionally, we note the possibility of using pharmacologically active substances that target various pro-oxidative and pro-fibrotic pathways found in chronic renal damage including hyperglycemia, mitochondrial energy dysregulation, mineralocorticoid hormonal signaling, and hypoxia-inducible factors (95). Ultimately, persistent fibrosis, progressive inflammation, and maladaptive renal repair compromise glomerular function, exacerbating toxin retention, oxidative imbalance, and the self-perpetuating cycle of renal decline (93).

### 4.3 Apoptosis and cell necrosis

Regarding cell death induced by OS, it is noted that, depending on the intensity of the stimulus, it can manifest itself in the form of apoptosis (controlled self-destruction of the cell in response to moderate OS, without generating major inflammation) or necrosis (uncontrolled cell death, generative of significant inflammation, in response to major OS) (96, 97).

Continuing from what was previously discussed, the transcription factors and essential pathways involved in the initiation and promotion of apoptosis and necrosis are the p53 factor, the mitogen-activated protein kinase pathways, NF-κB, and caspases. The first three regulate OS responses and promote apoptosis by modulating the expression of pro-inflammatory and pro-apoptotic genes. Caspases, however, serve a dual role, both supporting inflammation and inducing cell death by degrading cellular components in a controlled manner (96, 97). Functionally, caspases are classified as “initiator caspases” and “effector/executioner caspases”. Apoptosis can occur via two primary pathways: intrinsic (mitochondrial) or extrinsic (death-receptor-mediated), depending on the triggering mechanisms. The intrinsic apoptotic pathway is triggered by mitochondrial membrane permeabilization and damage. This pathway is regulated by B-cell lymphoma 2 family proteins and ROS-induced stress. These events result in the release of cytochrome c, exacerbation of OS, and disruption of ATP synthesis. Cytochrome c binds to apoptotic protease-activating factor-1, forming the apoptosome, a quaternary protein complex. The apoptosome then facilitates the recruitment and activation of inhibitor caspase-9, which subsequently activates effector caspases, leading to the controlled dismantling of cellular structures (96–99). The extrinsic apoptotic pathway is mediated by the death-inducing signalling complex, initiated by interactions between extracellular death ligands (e.g., Fas/CD95) and their respective cell surface receptors. This interaction activates intracellular death domains, recruiting Fas-associated protein with death domain (FADD) through a death domain interaction. FADD, via its death effector domain activates initiator caspase-8, which subsequently activates effector caspases (caspase-3, -6, and -7), culminating in apoptotic cell death (96, 99–101).

In contrast, necrosis resulting from severe OS starts by damaging the cell membranes. Lipid peroxidation, following the actions of ROS, induces membrane permeabilization in the early stages. In response to cytosolic calcium, intracellular enzymes (e.g., phospholipases, proteases) activate and damage membrane integrity by breaking down membrane and intracellular components. Later, the damage becomes irreversible, eventually leading to the rupture of the membrane with the release of its contents into the extracellular space. Among the components released at the extracellular level, we especially note damage-associated molecular patterns (DAMPs). DAMPs act predominantly on the immune system, inducing a strong inflammatory response that perpetuates the vicious cycle described in subchapter 4.2. and they exacerbate renal injury and accelerate NS progression. Additionally, DAMPs contribute to post-injury renal tissue regeneration and scarring (97, 99, 102–105).

At the mitochondrial level, ROS reduces the production of ATP and this is an important determinant of apoptosis versus necrosis susceptibility, affecting cellular functions such as sodium ion pump activity. Necrosis is promoted through cellular swelling (edema) and membrane depolarization due to calcium and water influx (97, 103, 106). More recently, literature has described regulated necrosis (necroptosis) as an intermediary process between apoptosis and necrosis, exhibiting a more structured and programmed nature. In this situation, the prototypical pathway is based on the interaction of TNF family members with specific ligands, the recruitment of receptor-interacting protein kinase 1, the inhibition of caspase 8, the assembly of the RIP1/RIP3 signaling complex, and the formation of the necrosome. In the necrosome, the phosphorylation of the mixed line kinase domain-like protein (MLKL) takes place, with its subsequent translocation to the plasma membrane. Here phosphorylated MLKL interacts with phosphatidylinositol phosphates, inducing its oligomerization and insertion at the membrane level. This culminates in cell lysis and the extracellular release of DAMPs. Additionally, phosphorylated MLKL activates the pyrin domain of the NOD-like receptor (NLR) family, specifically/including/and by extension inflammasome-3, promoting the secretion of pro-inflammatory cytokines IL-1β and IL-18, thereby perpetuating inflammation (103, 106–108).

**Figure 2:**
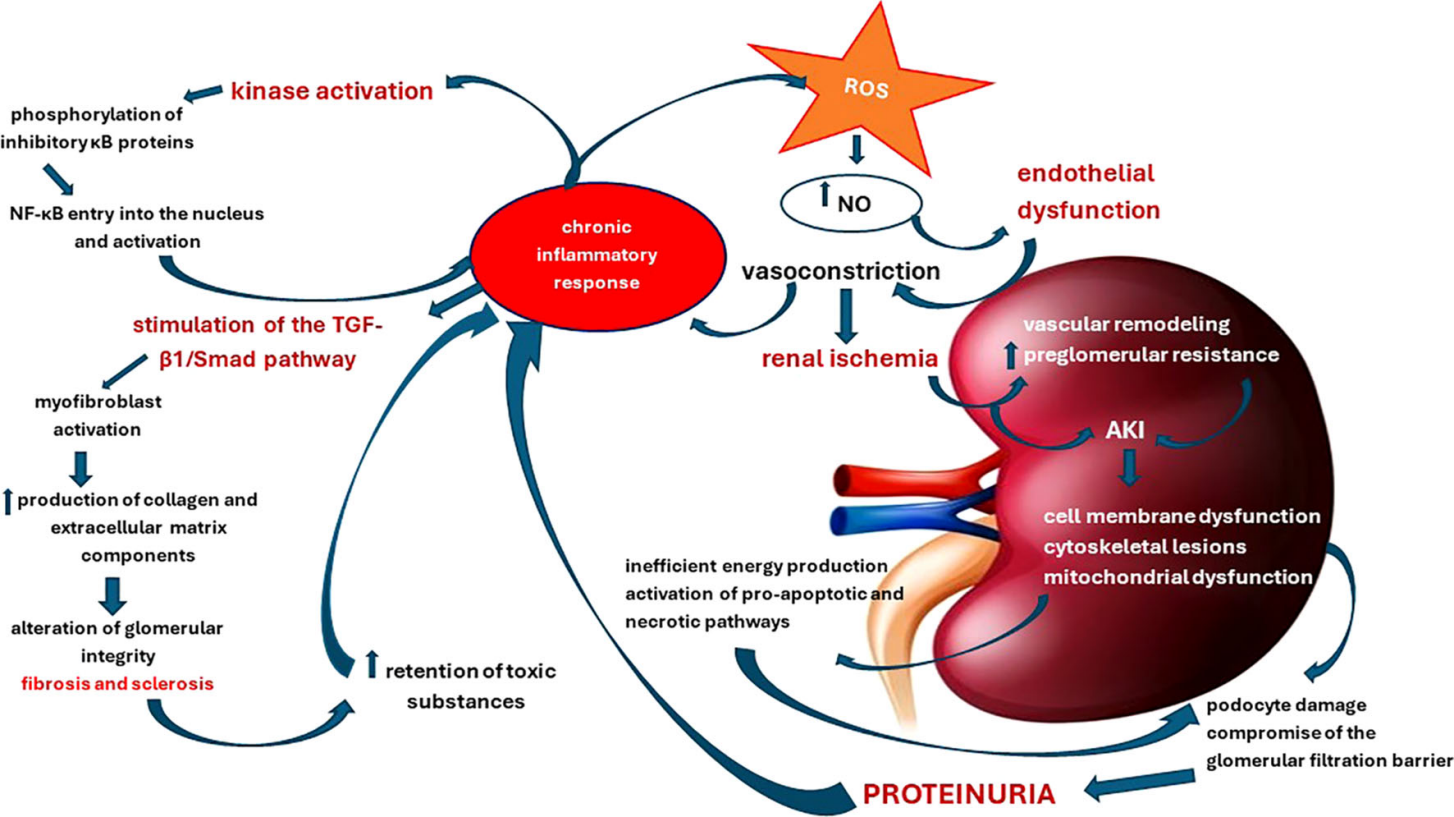
Pathophysiological cascade of renal structural and functional damage induced by oxidative stress and chronic inflammation.

As a consequence, in the dynamics of NS, the mechanisms of cell death – apoptosis and necrosis – operate in a complex way, being induced depending on the strength of the pro-oxidative stresses. These processes result in either cell proliferation or destruction of the glomerular barriers with varying degrees of inflammation and renal injuries that further exacerbate the course of the disease. In this intricate process, antioxidants have proven useful, with resveratrol emerging as an effective prophylactic and therapeutic agent due to its renoprotective properties. It has demonstrated its ability to directly or indirectly counteract the effects of ROS, as well as modulating key pathways such as NF-κB and maintaining mitochondrial energy balance (109). Finally, **Figure 2** illustrates how the increase in pro-oxidative status in NS initiates and maintains functional and structural renal decline.

### 4.4 Hyperlipidemia

Alterations in lipid metabolism, characterized by an increase in cholesterol, triglycerides, and low-density lipoproteins (LDL), are one of the major complications of NS that frequently go under-recognized. Although dyslipidemia is reported at a lower frequency in children than in adults, it acts as a significant contributor in the progression of both cardiovascular (e.g., atherosclerosis, thrombosis) and renal damage in affected individuals (110). Regarding the pathogenesis of NS-related dyslipidemia, extensive reporting has revealed impairments in the synthesis, plasma catabolism, and clearing of lipoproteins. These reports correlate the severity of hyperlipidemia with the degree of proteinuria, disturbances in enzyme activity following chronic tissue damage, and relapse. Additionally, dysfunction in hepatic, muscular, and adipose tissue exacerbates the risk of dyslipidemia by reducing the activity of hepatic lipase and lipoprotein lipase, while increasing hepatic levels of proprotein convertase subtilisin/kexin type 9, an enzyme involved in LDL receptor degradation (110–112).

OS appears to play a central role in dyslipidemia related to NS or chronic kidney disease, contributing to the worsening of proteinuria, hypoalbuminemia, and systemic inflammation. By these mechanisms, OS reduce blood enzyme activity. Its role in dyslipidemia is related to the oxidation of LDL, which occurs in this context of OS, marked by elevated ROS. Oxidized LDL contributes to dyslipidemia in two ways; first, increased amounts worsen the general systemic proinflammatory status and endothelial dysfunction. Secondly, as clearance is impeded during this pro-oxidative state, the concentration of circulating LDL becomes persistently high, increasing atherogenic risk. Additionally, OS disrupts liver activity, promoting increased insulin resistance, which in turn inhibits lipolysis in adipose tissue. This increases the level of free fatty acids taken up by the liver, and therefore the excessive production of LDL., At the renal level, the accumulation of excess lipid molecules causes cellular and mitochondrial damage, perpetuating the vicious cycle of renal dysfunction (113–116). Another component of the lipid balance affected in NS are high-density lipoproteins (HDL). We know that HDL is a protector against cardiovascular diseases, inflammation, and OS. However, in NS, HDL appears to undergo structural and functional changes which are reflected in a decreased HDL cholesterol-to-total cholesterol ratio and a reduced capacity to mature through esterification. These changes contribute to the deterioration of HDL-mediated reverse cholesterol transport, potentiating the atherogenic effects of proteinuria (116, 117). The studies carried out on the adult population indicated that supplementation with small doses of vitamin E was effective in protecting LDL from lipid peroxidation, thus promoting renal health (118). Other antioxidants useful in counteracting OS renal damage are N-acetyl cysteine, ascorbic acid, L-carnitine, coenzyme Q10, curcumin, and resveratrol (119).

### 4.5 Cardiovascular complications

Given that cardiovascular damage is less severe among children with NS than in adults, we shall briefly explore its implications. We believe that patient education, prevention, and optimal management of OS, systemic inflammation, and NS are key elements in reducing cardiovascular risk in children over the medium and long term. In support of this belief, Tkaczyk M. et al. argue that a severe clinical evolution of NS can be accompanied by an increased risk of accelerated atherogenesis. Also, persistent abnormalities of lipid metabolism may increase early atherogenic risk. In this sense, the current research is focused on defining the risk-to-benefit ratio of using lipid-lowering drugs in the pediatric population (73, 120).

As presented previously, we reiterate that NS is characterized by a persistent vicious cycle that emerges from the effects of OS, chronic pro-inflammatory status, hyperlipidemia, and metabolic imbalances. These components modulate each other, promoting and maintaining systemic damage by dysregulating the prooxidant/antioxidant balance. OS is important both in renal injury and subsequent cardiovascular-renal remodeling. Alongside renal dysfunction, OS and the activation of the renin-angiotensin system (RAS) represent key factors in the promotion of endothelial damage, arterial stiffening, atherosclerosis, arterial hypertension, and ventricular dysfunction. These comorbidities together may significantly impact NS-related morbidity and mortality. For example, it is known that atherosclerosis is important in determining the need for dialysis, kidney transplant outcomes, and mortality risk (121–123).

The biological regulation of homocysteine, inflammatory and prothrombotic blood factors, and adhesion molecules is one of the most important determinants in the earliest stages of atherosclerosis. Many of these factors are present in children with NS, either at initial presentation or during disease relapses. Literature links their presence to selective proteinuria, OS, and steroid therapy. An additional risk factor is renal replacement therapy, which exacerbates OS by accentuating the loss of antioxidant substances. OS-induced endothelial injury accelerates atherosclerosis, thereby increasing the risk of thrombotic events. This triad forms a critical bridge between nephrotic and vascular damage, ultimately leading to cardiac and cerebral damage of the ischemic type (72, 123). Supporting this theory, a correlation has been observed between urinary OS biomarkers and the arteriosclerosis index (123).

Regarding cardiac damage from a hemodynamic and clinical point of view, Kamel A. et al. reported that approximately 30% of children with NS exhibit right ventricular dysfunction, while 20% have left ventricular dysfunction. These impairments correlate significantly with disease duration, treatment history, relapse patterns, steroid resistance, and response to immunosuppressive medication (124). Similarly, Tharanidharan SP. et al. note an increase in the average myocardial performance index which was directly correlated to the frequency of relapses, disease duration, and a history of hypertension and hypercholesterolemia (115).

In summary, a self-perpetuating cycle underlies NS-associated cardiovascular damage, with OS at its core. OS severity itself is determined by various factors; among those that potentiate OS are carbohydrate metabolism dysregulation leading to advanced glycation end products, lipid profile abnormalities resulting in plasma lipid oxidation, immune system activation triggering proinflammatory cytokine release, renal hypoxia, and the adverse effects of steroid or immunosuppressant therapy. Meanwhile, antioxidant reserves are severely depleted in NS due to massive losses of plasma proteins, leading over time to a reduction in the body’s capacity to neutralize ROS. OS further perpetuates proteinuria, weakens antioxidant defense, sustains inflammation, and disrupts homeostasis. Additionally, it triggers RAS activation, promoting endothelial damage, arterial stiffening, atherosclerosis, arterial hypertension, and thrombosis. The ultimate cardiovascular consequences of this cascade include pathological cardiovascular remodeling, ventricular dysfunction, and ischemia (72, 73, 115, 120–124).

In managing the salient effects of OS on microvascular and renal homeostasis, we present the results obtained by Hertiš Petek T. et al. who highlight the benefits of lifestyle modifications, including regular physical exercise, reduction of sedentary periods, and an adequate diet, in improving insulin sensitivity while also enhancing antioxidant and anti-inflammatory capacity. The authors note the importance of maintaining a constant level of antioxidants in the plasma. Dietary supplementation with antioxidants (e.g., vitamin C, E, zinc, L-arginine, carnosine, phytophenols) or probiotics remains under research regarding its adjuvant therapeutic effects. One approach involves omega-3 polyunsaturated fatty acid supplementation, which has shown effectiveness in reducing the level of cytokines, ROS, and adhesion molecules. Other dietary strategies include carbohydrate-restricted and methionine-restricted diets (123). Currently, pharmacological interventions targeting the consequences of OS dominate clinical management strategies for cardiovascular complications in NS. We further urge the concentration of efforts in identifying and implementing adjuvant therapeutic schemes based on diets rich in antioxidants and/or their exogenous supplementation. We are confident that administering well-established antioxidant compounds with cardiovascular benefits will have the effect of reducing systemic inflammation, OS, and by extension cardiac damage by targeting pathophysiological cascades at three important levels: increasing systemic antioxidant capacity, reducing renal injury, and preventing disease relapses.

### 4.6 Effects on the immune system

NS in children, primarily in those with active diseases, is frequently accompanied by an increased risk of infection compared to the general population (125–127). Kalra S. et al. found that early detection of recurrence significantly reduced the number of patients experiencing severe infections (128). With regards to OS, immune disorders in pediatric NS () are related to disruptions in immune cell proliferation and function, urinary loss of immunoglobulins, and an imbalance in immune responses – suppression of cellular immunity (low Th1) and an excessive stimulation of the humoral response (high Th2). This imbalance promotes the secretion of TNF-α, IL-6, IL-8, and IL-1β, complement activation, and neutrophil recruitment, which further increases the production of free radicals, amplifying the vicious cycle of proteinuria, podocyte damage, inflammation, and OS.

At the same time, corticosteroid therapy induces lymphocyte apoptosis and weakens the body’s capacity to combat viral and bacterial infections, as well as compromising the barrier function of immune cells following lipid peroxidation and cell membrane damage (121). While the urinary losses of functional proteins have been previously detailed, we turn our attention now to the damage inflicted on immune cells in NS. Once again, excessive concentrations of ROS are implicated among other reasons. Though physiological levels of ROS play a crucial role in immune function, excessive amounts have shown to impair the viability and function of immune cells, reducing the body’s ability to counteract infections. Furthermore, infections exacerbate inflammation, tissue damage, and metabolic disturbances, all of which sustain OS, Therefore, targeted OS management through antioxidant therapies and ExAs (e.g., α-tocopherol, ascorbic acid, coenzyme Q10, N-acetylcysteine, resveratrol, selenium, zinc, and curcumin) appears to be a reliable treatment approach to improving immune resistance in patients with NS. However, in order not to produce more harm than good, we must aim to restore ROS to physiological levels rather than eliminating them entirely (129–131).

Another mechanism contributing to immune dysregulation and increased relapse susceptibility in NS is intestinal dysbiosis. Alterations in intestinal microbiota have been shown to impair inflammatory cell function by disrupting the Th17/Treg axis following the depletion of butyrate- or short-chain fatty acid-producing bacteria. Furthermore, dysbiosis has also been shown to correlate with the stages of NS. Impaired microbiota function has clear implications in the pathogenesis of NS, linking intestinal dysbiosis with Treg cell dysfunction and increased relapse risk. In accordance with the above, clinical trials have demonstrated that modulation of the gut microbiota by administering probiotics based on butyrate-producing bacteria led to a longer relapse interval and a reduced need for immunosuppressive agents. Also, treatment with prebiotics and probiotics in addition to steroids demonstrated a significant increase in T-reg cells (CD4+/CD25+/FOXp3+) in peripheral blood and a higher level of Lactobacillus species in stool, along with a significant decrease in relapse rate (132–136).

### 4.7 Edema and electrolyte imbalances

OS encountered in pediatric NS triggers and sustains a pathophysiological cascade that progressively worsens glomerular lesions, proteinuria, and hypoalbuminemia. Additionally, studies in murine models have demonstrated the critical role of ROS in hydro-electrolyte disturbances associated with NS. OS primarily affects three key mechanisms: (1) up-regulation of Na/K-ATPase, (2) increases water permeability while simultaneously reducing the protein reflection coefficient through the peritoneal barrier, and (3) stimulation of angiotensin-induced aldosterone secretion (137). Also, salt overload (e.g., in the case of a high-sodium diet) has been shown to promote OS by modulating key molecules in immunity and inflammation, thereby perpetuating electrolyte imbalances, endothelial damage, and the progression of renal disease (138–140). The results of these systemic-related mechanisms contribute to the formation of edema characteristic of NS (141). However, some studies implicate the epithelial sodium channel activated by proteolytic enzymes or their precursors in sodium retention and subsequent edema, in particular cases (142–144). Therefore, the mechanism of edema production in renal diseases is complex, involving several intricate factors, among which OS plays a significant role.

### 4.8 Response to corticosteroids

OS, an important element in NS, may appear to modulate treatment response. The intensity and duration of OS exposure appear to correlate with corticosteroid responsiveness,. Assessing total antioxidant capacity can be useful in predicting the response to corticosteroids at diagnosis or relapse after the interruption of steroid treatment (145). In contrast to other chronic inflammatory diseases such as asthma or chronic lung disease, the mechanisms underlying steroid sensitivity variability in NS remain unclear. However, a disturbed redox environment (marked by decreased levels of enzymatic and non-enzymatic substances with antioxidant roles) affects glucocorticoid receptor sensitivity and immune cell function. There are many interesting areas of research that can be explored, such as the impact of OS on the structural conformation of corticosteroid receptors, intracellular signaling pathways, renal cell integrity, or hormonal dynamics,. Excessive ROS may interfere with glucocorticoid receptor degradation and translocation, affecting their activity. At the same time, persistent inflammation and chronic activation of NF-κB can lead over time to decreased sensitivity to steroids. Finally, ROS can activate mitogen-activated protein kinase which further interferes with glucocorticoid receptor signaling by phosphorylating and reducing its ability to bind to DNA and regulate anti-inflammatory genes. To mitigate this damage, antioxidants known to specifically target the oxidative pathways can be used such as N-acetylcysteine, glutathione precursors, vitamins C and E, curcumin and other NF-κB inhibitors, and mitochondria-targeted antioxidants such as MitoQ. Of these substances, the most used in practice will be detailed below (146–150).

## 5 Reliable biomarkers in the assessment of the oxidative status and prognosis of NS

Considering the multiple implications of OS in the dynamics of NS, we will discuss the main biomarkers at the systemic level, as well as the utility of renal prognosis. For an easier understanding, biomarkers can be categorized as enzymatic and non-enzymatic (stable ROS attack products). Their clinical value lies in enabling disease monitoring, guiding therapeutic adaptation, prevention of relapses, and improving medium and long-term prognosis. A notable advantage of oxidative biomarker assessment is its non-invasiveness. However, selecting relevant biomarkers requires a critical analysis of specificity, measurement site, and technique, as individual variations have been reported. The practical challenges of implementing biomarker-based monitoring strategies include being unable to reliably and clinically predict NS progression, lack of standardized measurement methods, and the need for advanced equipment and financial resources, which may limit accessibility in public healthcare systems. At the same time, we recognize that results must be interpreted in the context of genetic factors, diet, comorbidities, and concurrent treatments,. Large-scale clinical trials are needed to establish the real benefits of biomarker-based, personalized treatments in NS. Although there are challenges in achieving consensus within the medical community and updating treatment guidelines accordingly, personalizing treatment based on OS biomarkers could represent an important step in improving NS management, optimizing therapies, and reducing disease progression (151, 152).

Enzymatic biomarkers primarily include antioxidant enzymes such as superoxide dismutase, catalase (CAT), and glutathione peroxidase. These enzyme systems intervene in the dismutation of superoxide anion free radicals into molecular oxygen and hydrogen peroxide, the reduction of hydrogen peroxide and lipid hydroperoxides. In this way, antioxidant enzyme systems protect cells from damage induced by ROS attacks. During these processes, optimal enzyme activity depends on essential cofactors (e.g., copper, zinc), and their reduced activity signals increased OS, and compromised antioxidant defense (153–157). Additionally, it is possible to evaluate the degree of OS by measuring the ratio between reduced glutathione and oxidized glutathione (158). Non-enzymatic biomarkers of NS-specific OS include advanced protein oxidation products, malondialdehyde (a lipid peroxidation marker), 8-hydroxydeoxyguanosine (a marker of oxidative DNA damage), and total antioxidant capacity (TAC). In contrast to TAC, whose low level is associated with redox system dysfunction, the other biomarkers are associated with OS at increased values (159, 160).

From a prognostic perspective, patients with a lower-than-normal antioxidant status experience greater OS, such that they are more likely to suffer from unfavorable prognostic outcomes, for example, increased risk of relapse and quicker degeneration of kidney tissues. Hence, the integration of enzymatic and non-enzymatic biomarkers into NS treatment strategies may enable personalized therapy. Emerging research focuses on CAT gene polymorphism and their role in steroid resistance in idiopathic NS (161, 162). In practical terms, we have found studies that associate OS biomarkers and antioxidant potential with NS progression, relapse frequency, and renal failure (163–166).

## 6 Means of manipulating antioxidant capacity

Although the progression to chronic renal failure is rarer in children than in adults, the effective management of OS remains crucial, given its role in systemic decline,. Controlling the pro-oxidative status allows clinicians to reduce the morbidity associated with long-term complications of the underlying disease (e.g., cardiovascular and metabolic diseases in adult life) (167).

As previously discussed, antioxidants involved in combating NS-specific OS can be categorized as enzymatic and non-enzymatic. Enzymatic antioxidants rely heavily on endogenous production and functionality, necessitating optimal cofactor levels through exogenous intake (54, 83, 155). In contrast, non-enzymatic antioxidants, primarily obtained from external sources (e.g., vitamin C and E, coenzyme Q, carotenes, and trace elements), play roles in neutralizing ROS, oxidation prevention, and enzymatic function. However, certain non-enzymatic antioxidants – such as glutathione, ubiquinone, albumin, metallothioneins, and uric acid – are predominantly produced endogenously (28–30, 155, 168).

Antioxidant supplementation, whether dietary or pharmacological, is used as an adjuvant therapeutic method alongside standard steroid or immunosuppressive treatments. It can have various homeostatic benefits with regards to the systemic response to oxidative aggression, and these benefits depend on individual substance properties (e.g., bioavailability, synergy). Therefore, understanding the physicochemical characteristics, mechanisms of action, and interactions of ExAs is essential for optimizing bioavailability and bioactivity. Additionally, we especially emphasize the usefulness of antioxidants as adjunctive therapy, complementary to lifestyle changes (e.g., diet based on fruits, tea, vegetables or cereals) and targeted pharmacological interventions. The most effective approach integrates dietary changes, conventional pharmacological therapy, and antioxidant supplementation, as supplementation alone cannot counteract an unhealthy lifestyle (28–30, 155, 167, 168).

We reiterate that in the case of NS, chronic biological disturbances cause fluctuations in antioxidant levels, a phenomenon also observed in the case of infectious processes related to the activation of systemic inflammatory pathways. Since NS is a chronic pathology with a fluctuating evolution and multiple intercurrents, continuous OS management is important for reducing morbidity, slowing renal disease progression, minimizing relapses, and optimizing treatment response. To reduce OS, three levels must be targeted: decreasing exposure to environmental pollutants with oxidizing properties, increasing the administered level of EAs and ExAs, and reducing OS generation. Long-term lifestyle strategies must be carefully developed through cooperation within multidimensional teams that integrate both pharmacologists and dieticians. These strategies may include antioxidant supplementation, pharmacological agents with antioxidant properties (e.g., statins), and strict inflammation control. Importantly, care must be taken not to create a pro-oxidative environment through the excess administration of corticosteroids., Adopting an antioxidant-rich diet with a high content of polyphenols and flavonoids, reducing salt and unsaturated fat consumption, and ensuring adequate protein intake can mitigate OS, reduce cardiovascular complications, and prevent malnutrition. Psychological stress can act as a relapse trigger and should be managed with relaxation techniques and moderate physical exercise., Given their role in the pathophysiological cascade of NS, there is a growing interest in studying and integrating OS biomarkers into clinical practice for individual personalized therapy. Current research is focused on identifying and optimizing antioxidant supplementation strategies to reduce NS-related morbidity (134, 163, 169–171). The key related approaches are summarized in **Table 3**.

**Table 3:** Exogenous antioxidants with therapeutic potential in NS (75, 78, 90, 119, 123, 172–176).

Substance	Description	Function	Source
Taurine	improves the urinary excretion of proteins, the generation of reactive species at the mitochondrial level and podocyte apoptosis.	improves the biological status and the renal histopathological appearance.	meat, fish, beef, chicken, dairy products, eggs.
Melatonin	reduces podocyte apoptosis and improves their proliferative capacity.	protective effect against oxidative stress, favoring the recovery of mitochondrial function.	cherries, goji berries, eggs, dairy products, fish, pistachios, grapes, corn.
L-arginine	improves endothelial dysfunction by increasing nitric oxide bioavailability in animal and human models without chronic kidney disease.	reduces the endothelial dysfunction responsible in part for the progression of renal aggression and chronic cardiovascular complications.	meat, poultry, dairy, nuts, soy products, and fish
L-carnitine	protects the cell membrane structures, increases the level of glutathione and the activity of glutathione peroxidase, in parallel with the reduction and decreases the level of malondialdehyde.	protects cellular structures from the negative effects of prolonged pro-oxidative status. Improves antioxidant capacity.	red meat
Folic acid	inhibits oxidative stress and apoptosis of podocytes, independently or by modulating the plasma level of homocysteine.	attenuates glomerular lesions.	brussels sprouts, kale, spinach, fortified bread and breakfast cereals, broccoli, cabbage, cauliflower, chickpeas, green beans, icebergs, lettuce, kidneys, beans, peas, spring greens, potatoes, most other vegetables, most fruits, most nuts, brown rice, wholegrain pasta, oats, bran, some breakfast cereals, cheese, yoghurt, milk, eggs, salmon, beef, game.
Alpha-lipoic acid	reduces reactive oxygen species, participates in the recycling of other antioxidants in the body (e.g. vitamins C and E and glutathione) and protects against protein and lipid oxidation.	improves the pro-oxidative status, potentiates the action of other antioxidant substances, improves the decline of renal function and endothelial dysfunction.	spinach, broccoli, tomatoes, Brussels sprouts and rice bran, meat and organs.
Zinc	improves levels of apolipoproteins A1 and B, oxidized low-density lipoproteins, leptin, malondialdehyde and C-reactive protein, while decreasing markers of insulin resistance.	decreases the risk of cardiovascular damage (atherosclerosis and coronary heart disease).	lamb, leafy and root vegetables, crabs and shellfish, beef, offal, whole grains, pork, poultry, milk and milk products, eggs, nuts.
Coenzyme Q10	improves mitochondrial function, reduces total cholesterol, LDL cholesterol, lipid oxidation, malondialdehyde and creatinine; no effect on resting blood glucose, insulin resistance and inflammatory markers.	decreases the risk of cardiovascular damage.	meat, fish, nuts, and some oils, dairy products, vegetables, fruits, and cereals.
Vitamin E (alpha-tocopherol)	protects against lipid peroxidation, increases resistance to low-density lipoproteins, regulates inflammatory gene expression.	reduces the progression of kidney damage, cardiovascular complications and chronic inflammatory status.	wheat germ oil, almonds, sunflower seeds and oil, safflower oil, hazelnuts, peanuts and peanut butter, corn oil.
Vitamin C	- prevents oxidative damage by directly scavenging superoxide anion and hydroxyl radical. - participates in the negation of other antioxidant substances (e.g., vitamin E).	- reduces the progression of kidney damage, cardiovascular complications and chronic inflammatory status. - simultaneous administration of vitamin C and vitamin E decreases the formation of carbonyl compounds and the concentration of malondialdehyde, in parallel with the increase of the total antioxidant capacity.	kiwi fruit, citrus fruit (oranges, lemons, satsumas, clementines, etc.), black currants, guava, mango, papaya, pepper, brussels sprouts, broccoli, sweet potato.
Vitamin B	inhibits the formation of advanced glycation end products.	decreases selective proteinuria (albuminuria) and disease progression.	turkey meat, dairy products, eggs, yogurt, potatoes, carrots, green beans, eggplant, peas, broccoli, mushrooms and kale, cantaloupe, raspberries and sunflower seeds, peanuts, walnuts, wheat germ, whole grains.
Vitamin D	reduces oxidative stress and inflammation by modulating total antioxidant capacity, glutathione, malondialdehyde, nuclear factor erythroid 2-related factor 2 and regulation of antioxidant enzymes.	reduces renal and cardiovascular damage, a certified aspect by reducing proteinuria and improving endothelial cardiovascular markers.	cod liver oil, oily fish (salmon, mackerel, etc.), milk, margarine, breakfast cereals, eggs, liver.
Polyunsaturated fatty acids	anti-inflammatory properties, intervening in reducing the level of interleukins, tumor necrosis factor and C-reactive protein.	reduce systemic inflammation.	eggs and dairy products, fish oils, rapeseed oil, olive oil, fruit, vegetables, beans, and pulses.
Quercetin, resveratrol, curcumin, carnosine, sage, berberine, apigenin, geniposide, polyphenols, procyanidin B2	negatively regulates pro-oxidative enzymes, increases the activity of the mitochondrial respiratory chain, increases the activity of endogenous enzymatic and non-enzymatic antioxidants, modulates apoptotic pathways mediated by oxidative stress.	anti-inflammatory, anti-apoptotic and anti-fibrotic effects.	green tea, grapes, pomegranate.

## 7 Conclusions

There is a strong association between systemic OS and the progression of pediatric NS,. The relationship is bidirectional; excess ROS participates through various pathophysiological mechanisms in the onset and persistence of renal damage and its complications – cardiovascular diseases, hydro-electrolytic and acid-base imbalances, immune dysfunction, dyslipidemia, and disease progression - while renal and systemic homeostasis disruption perpetuates a pro-inflammatory state, toxic substance retention, and a variety of metabolic abnormalities, in turn fueling OS. However, pharmacotherapy for NS, as well as other exogenous toxicants, are also implicated in OS etiology.

This review intended to clearly and comprehensively summarize the key pathophysiological mechanisms underlying renal and systemic deterioration in pediatric NS from an OS perspective. Our practical purpose is to demonstrate and encourage the use of antioxidant-rich diets and, non-enzymatic antioxidant supplementation in interrupting the pathophysiological cascade of NS. Implementing these strategies may help prevent or mitigate local and systemic complications over the medium and long term while reducing disease-specific morbidity at minimal cost and invasiveness.

We also emphasize the potential of enzymatic and non-enzymatic biomarkers as diagnostic and prognostic tools. Finally, we advocate for direct global efforts towards the development and patenting ofmethods for assessing oxidant and antioxidant status in pediatric clinical practice, particularly for chronic disorders characterized by low-grade chronic systemic inflammation. We believe that they will offer clinicians novel prospects for the personalized, diagnostic, and therapeutic management of pediatric NS, resulting in significant reduction of morbidity and mortality.
